# High Total Bilirubin as a Protective Factor for Diabetes Mellitus: An Analysis of NHANES Data From 1999 - 2006

**DOI:** 10.4021/jocmr425w

**Published:** 2010-10-11

**Authors:** Pramil Cheriyath, Venkata Subhash Gorrepati, Ian Peters, Vinod Nookala, Megan E Murphy, Nadine Srouji, Daniel Fischman

**Affiliations:** a Pinnacle Health/ Harrisburg Hospital, Harrisburg, PA, USA; b Philadelphia College of Osteopathic Medicine, Philadelphia, PA, USA

## Abstract

**Background:**

Diabetes Mellitus (DM) is a rampantly growing epidemic in the United States, affecting nearly 10% of the adult population. Studies have shown that higher levels of Total Bilirubin (TBili) convey a protective effect with regard to cardiovascular risk. In this study, we will examine the relationship between TBili level and prevalence of DM to discern whether a similar relationship exists.

**Methods:**

The National Health and Nutrition Examination Survey (NHANES) is a comprehensive survey performed regularly to evaluate the overall health and nutrition status of the United States population. For the purpose of this study, we combined NHANES data collected between 1999 and 2006. Totally 15,876 eligible participants were selected after excluding all patients younger than twenty years, those with a history of abnormal liver function tests, or those who disclosed a history of liver disease. The data collected on these individuals was adjusted for demographic characteristics, as well as risk factors for DM, and was analyzed via multivariate logistic regression, using SAS proc survey methodology.

**Results:**

After age adjustment, increased TBili was associated with 26% reduction in diabetes risk (OR 0.74, 95% CI 0.64 - 0.88). Multivariate analysis, adjusting for all diabetes risk factors assessed, confirmed this association (OR 0.80, 95% CI 0.67 - 0.95).

**Conclusions:**

Our results show that a higher level of serum TBili is associated with odds of having a lower incidence of DM. This finding supports the hypothesis that the antioxidant nature of TBili, demonstrating a protective effect with regard to the risk of stroke, atherosclerosis, and vasculitis in prior research, also extends to DM risk. Furthermore, research has shown that higher levels of TBili increase glucose mobilization into the cells, leading to more efficient, biologic glucose utilization. There is no doubt that the beneficial effect of TBili is multifactorial; thus further investigation is warranted.

**Keywords:**

Bilirubin; Diabetes; Antioxidant; Protective

## Introduction

Diabetes Mellitus has become an epidemic throughout the world. Prevalence data, collected in 2007, indicate that 7.8% of the population have been diagnosed with this disease [1]. From a fiscal standpoint, the diagnosis of Diabetes Mellitus is associated with a two-fold rise in medical cost, equaling approximately $116 billion dollars in direct expenditures and $58 million in indirect expenditures, in the United States (US) [1]. Given this disease’s dramatic impact on the physical and financial health of the US population, disease prevention has taken on paramount importance. With this focus in mind, research activity has focused on finding physiologic factors that decrease a person’s risk of developing Diabetes Mellitus.

Bilirubin has been conjectured to be one such factor. There is a growing body of literature which shows that higher Total Bilirubin levels (TBili) are protective against cardiovascular disease, Stroke and Peripheral Arterial Disease [2-7]. Studies suggest that an increased expression of Heme Oxygenase, an enzyme used to break down the hemoglobin into bilirubin, is associated with enhanced insulin sensitivity and glucose metabolism, thus resulting in greater rates of rat model euglycemia [8, 9]. Furthermore, the antioxidant properties of TBili have been postulated to reverse oxidative damage associated with a hyperglycemic state [10]. Consequently, we have undertaken this population-based study to assess whether higher levels of TBili, within the physiologically normal range, convey a decreased risk of Diabetes Mellitus in the US population.

## Materials and Methods

The National Health and Nutrition Examination Survey (NHANES) is a nation-wide survey performed jointly by the National Center for Health Statistics and the Center for Disease Control and Prevention (CDC), on a representative sample of the US population. This sample is chosen using a stratified, multistage probability cluster sampling design of the non-institutionalized, non-military US population [11]. Components of this survey include questionnaires about demographics and health status, general medical examinations, as well as laboratory tests of varied bodily fluids. The physical examination component of NHANES consists of medical, dental, AND anthropometrical measurements collected by trained personnel [12].

For the purpose of our study, we examined NHANES data collected between 1999 and 2006. Exclusion criteria in our protocol removed all data for NHANES participants who were younger than twenty years-of-age, who reported a history of liver disease, who had abnormal liver function studies, or who had missing data values. Of 41,474 participants who had data collected during our seven-year period-of-interest, 15,876 did not meet any of the exclusion criteria. Based on previous studies we stratified TBili into two groups. One had the TBili level of less than 10 micromol/L and the other had a level of greater than or equal to 10 micromol/L [2, 23].

### Laboratory methods

To measure Total Bilirubin, NHANES used the LX20 process which utilizes a timed endpoint Diazo method. The basis of this method is formation of Azobilrubin from a reaction with a Diazo reagent. The change in the absorbance level of the reagent is measured, as this value is directly proportional to the concentration of Bilirubin. Fasting blood sugar was measured using Roche/Hitachi 911 instrument (Roche Diagnostics, Indianapolis) on early morning serum sample, for which subjects were asked to fast for nine hours. [11].

### Case definition

Case definition of Diabetes Mellitus was based on fasting blood sugar value of not less than 126 mg/dl (7.0 mmol/L) and the participant’s response to the following questions: (1) ‘Other than during pregnancy, have you ever been told by a doctor or other health professional that you have diabetes or sugar diabetes?’; (2) ‘Are you taking insulin now?’; (3) ‘Are you taking any pills to decrease your blood sugar?’.

### Definition of covariables

The NHANES questionnaire section was used for the characterization of age, sex, race, tobacco use pattern, alcohol use, highest level of education achieved, and marital status. Hypertension was defined as a mean systolic blood pressure of 140 mm Hg, a mean diastolic blood pressure of 90 mm Hg, or reporting a physician diagnosis of high blood pressure. Total cholesterol was defined as reporting that a physician had diagnosed that person with high cholesterol or reporting that a physician had advised that person to take cholesterol lowering medications. Prevalent cardiovascular disease was defined as a self-reported history of coronary heart disease, previous heart attack, or history of stroke. BMI was categorized into the three categories of normal, overweight and obese based on CDC guidelines [13].

### Statistical analysis

In order to account for the complex, stratified, multistage probability cluster sampling design employed in the NHANES survey, we analyzed this data using SAS version 9.1’s (Cary, North Carolina) Proc Survey methodology. Variance was estimated using the jackknife replacement method. We calculated the age-adjusted prevalence estimates, stratified by demographics and pre-determined risk factors. We used proc survey logistic regression to create a risk-factor based model, and calculated odds ratios with 95% confidence Intervals. These models were specifically adjusted for age, sex, race, smoking, alcohol, education and marital status.

## Results

**Figure 1.**
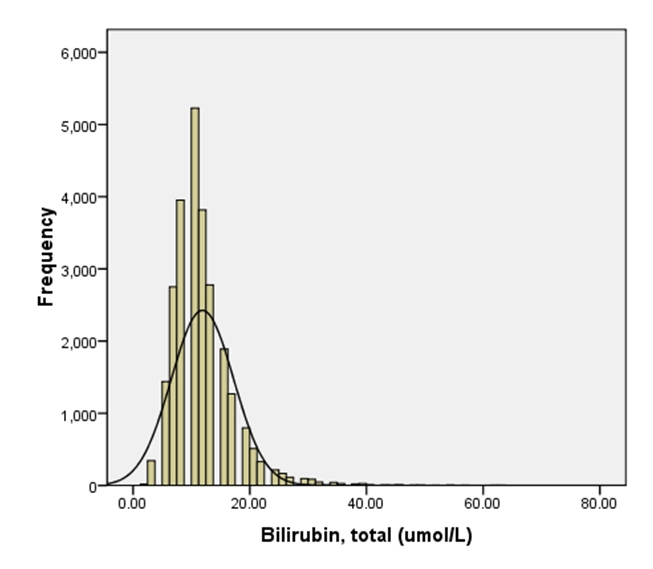
Distribution of Bilirubin (TBili) levels in the target population.

The bilirubin levels in the sample population were normally distributed, with a small right sided tail (Fig. 1). The mean TBili level was 11.89 micromol/L, with a standard deviation of 5.31.

**Figure 2.**
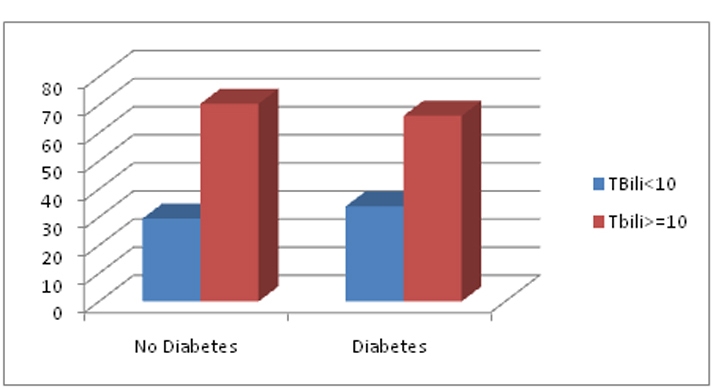
Prevalence of TBili values in patients with Diabetes and No Diabetes.

**Figure 3.**
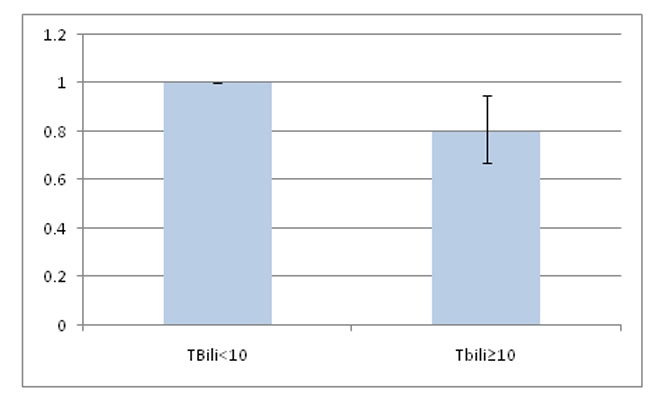
Comparing the adjusted odds ratio of having Diabetes in TBili < 10 and Tbili ≥ 10 groups.

The demographic variables of the two groups were compared by age adjusted prevalence (Table 1). A greater proportion of men were found to have a serum TBili level above 10 micromol/L. For individuals who had Diabetes Mellitus, 70.45% (SE = 0.86) had a TBili level of 10 micromol/L. Furthermore, a statistically-significant, unadjusted association between higher TBili level and Diabetes did exist (OR: 0.74, 95% CI: 0.64 - 0.88). The relationship remained significant, after adjusting for age, sex, race, married status, education, BMI and smoking (OR: 0.80, 95% CI: 0.67 - 0.95) (Fig. 2 and 3).

**Table 1:** Demographic Characteristics (Age Adjusted Prevalence)

	Serum TBili < 10 micromole/liter	Serum TBili ≥10 micromole/liter
Age 20 - 39	32.28% SE 1.18	67.72% SE 1.18
40 - 59	28.93% SE 0.93	71.07% SE 0.93
60 and above	27.43% SE 1.15	72.57% SE 1.15
Male	17.83% SE 0.80	82.16% SE 0.80
Female	40.45% SE 1.04	59.55% SE 1.04
Edu < High school	37.09% SE 1.39	62.91% SE 1.39
Edu > High school	28.21% SE 0.81	71.79% SE 0.81
Non-black	28.82% SE 0.86	71.18% SE 0.86
Black	39.01% SE 2.01	60.94% SE 2.01
Non smoker	28.51% SE 0.95	71.49% SE 0.95
Smoking	31.34% SE 1.02	68.66% SE 1.02
No alcohol consumption	28.98% SE 1.20	71.02% SE 1.20
Moderate alcohol consumption	28.36% SE 1.16	71.64% SE 1.16
Heavy alcohol consumption	32.12% SE 1.02	67.89% SE 1.02
Low triclycerides	28.87% SE 0.79	71.13% SE 0.79
High triclycerides	32.13% SE 1.23	67.87% SE 1.23
Lower waist circumference	25.52% SE 0.86	74.48% SE 0.86
Higher waist circumference	34.30% SE 1.05	65.70% SE 1.05
BMI less than 25	28.14% SE 1.01	71.86% SE 1.01
25 - 29	27.11% SE 1.02	72.89% SE 1.02
30 and above	35.00% SE 1.21	65.00% SE 1.01

## Discussion

Our study supports the notion that people with high TBili have lesser odds of prevalence of Diabetes Mellitus (DMI and DMII). This is in keeping with previously reported studies which have shown higher TBili levels to be protective against Coronary Artery Disease, Stroke and Peripheral Vascular Disease [2-7].

Bilirubin has been regarded as a powerful endogenous anti-oxidant and anti- inflammatory agent [14-18]. Thus, it stands to reason that higher TBili levels may be protective against the autoimmune, inflammation-related pathology of type 1 diabetes and oxidative physiologic stress associated with development of type 2 diabetes [19]. Current research also suggests that physiological levels of TBili block the production of various free radicals that might hinder the inhibitory responses of the cell to take up the high glucose [20]. Furthermore, it is shown to prevent the vascular endothelial activation from the oxidative stress in the vessels [21, 22].

In addition, rat model studies of DM II have shown that increased expression of Heme Oxygenase-1, the enzyme responsible for conversion of Hemoglobin to Bilirubin, is associated with enhanced insulin sensitivity and glucose metabolism resulting in greater rates of rat model euglycemia. Moreover, Hemin, an activator of the Heme Oxygenase system, has been found to increase the expression of GLUT-4 receptors and Adiponectin, a hormone which increases insulin sensitivity [20]. Similar to TBili, and in light of its anti-inflammatory properties, higher Adiponectin levels have been associated with lower DM prevalence. However, unlike Adiponectin since TBili is a commonly measured laboratory value, measuring TBili levels would offer a cost-effective method of DM risk stratification.

This study follows a complex sampling with the provision of the weights, which makes it more realistic to project on to the United States population. The amalgamation of four large cross sectional surveys done on the representative population of the United States from 1999 to 2006 translated this study high volume population based study. So we got a fair amount of population who have diabetes and who didnt. The stratification of total bilirubin into 2 groups, one having a TBili level of less than 10 micromol/L and the other having the TBili level of greater than or equal to 10 millimol/L is based on previous study which showed that a level of TBili equal to or above 10 millimol/L is cardio protective [23].

Another interesting observation is that there is a greater distribution of population in the higher TBili group which had the bilirubin greater or equal to 10 micromol/L, which was nearly consistent in all demographic groups. Needless to say also there is most of the population distribution in the latter group (≥ 10 micromol/L) with respect to prevalence of diabetes and prevalence of ‘no diabetes’ (66.1% vs 33.8% of diabetes group and 70.5% vs 29.5% of ‘no diabetes’ group.

Our use of population-adjusted data from a national survey, using predetermined definition is not without its limitations. One major limitation to this study is that the definition of Diabetes Mellitus is predicated on subject recollection and self reported diagnosis, thus introducing a component of recall bias. In order to compensate for this, we have added fasting blood sugar measurements into our study’s definition of Diabetes Mellitus.

For similar reasons, our study is limited in its ability to determine whether the protective effects of TBili are limited to only one form of Diabetes Mellitus, or whether the protective effect influences the incidence and prevalence of both DM type1 and type 2. Also, the cross sectional nature of the data derived from NHAHES’s methodology hinders determination of a temporal association or the cause-and-effect between TBili and development of DM type 1 or type 2. The lack of fractionation of TBili also limits our ability to determine whether it is the indirect fraction, the direct fraction, or a combined effect of the varied fractions of TBili, which convey the protective effect we described in this study [24].

In conclusion, there appears to be a beneficial effect of TBili on Diabetes. Further investigation is warranted in order to delineate whether this protective phenomenon extends to all forms of Diabetes Mellitus, whether it is conveyed by both Indirect and Direct Bilirubin, and what avenues are available to therapeutically raise an at-risk patients bilirubin levels for optimal protection from this dreaded disease.
